# Supplementary material for: A Critical View of the Application of the APEX Software (Aqueous Photochemistry of Environmentally-Occurring Xenobiotics) to Predict Photoreaction Kinetics in Surface Freshwaters
Source: Molecules. 2019 Dec 18;25(1):9. doi: 10.3390/molecules25010009 (PMC7017383; doi:10.3390/molecules25010009)
Supplement: Supplementary file 1 [file molecules-25-00009-s001.zip › 3.2.4_gcc-4.4.0.zip!/3.2.4_gcc-4.4.0/3.2.4_gcc-4.4.0/doc/octave/html/liboctave/Quadrature.html]

Quadrature - Octave C++ Classes


Next: Ordinary Differential Equations,
Previous: Optimization,
Up: Top


---

## 10 Quadrature

— : **Quad** (integrand\_fcn fcn)  
— : **Quad** (integrand\_fcn fcn, double abs, double rel)

— : virtual double **integrate** (void)  
— : virtual double **integrate** (int &ier)  
— : virtual double **integrate** (int &ier, int &neval)  
— : virtual double **integrate** (int &ier, int &neval, double &abserr) = 0

— : **Quad\_options** (void)  
— : **Quad\_options** (const Quad\_options &opt)

— : Quad\_options& **operator =** (const Quad\_options &opt)

— : void **init** (void)

— : void **copy** (const Quad\_options &opt)

— : void **set\_default\_options** (void)

— : void **set\_absolute\_tolerance** (double val)

— : void **set\_relative\_tolerance** (double val)

— : double **absolute\_tolerance** (void)  
— : double **relative\_tolerance** (void)

— : **DefQuad** (integrand\_fcn fcn)  
— : **DefQuad** (integrand\_fcn fcn, double ll, double ul)  
— : **DefQuad** (integrand\_fcn fcn, double ll, double ul, double abs, double rel)  
— : **DefQuad** (integrand\_fcn fcn, double ll, double ul, const ColumnVector &sing)  
— : **DefQuad** (integrand\_fcn fcn, const ColumnVector &sing, double abs, double rel)  
— : **DefQuad** (integrand\_fcn fcn, const ColumnVector &sing)  
— : **DefQuad** (integrand\_fcn fcn, double ll, double ul, const ColumnVector &sing, double abs, double rel)

— : **IndefQuad** (integrand\_fcn fcn)  
— : **IndefQuad** (integrand\_fcn fcn, double b, IntegralType t)  
— : **IndefQuad** (integrand\_fcn fcn, double b, IntegralType t, double abs, double rel)  
— : **IndefQuad** (integrand\_fcn fcn, double abs, double rel)

- Collocation Weights
